# Prevalence, associated factors, and disclosure of intimate partner violence among mothers in rural Bangladesh

**DOI:** 10.1186/s41043-020-00223-w

**Published:** 2020-12-07

**Authors:** Stephen Stake, Saifuddin Ahmed, Wietse Tol, Salahuddin Ahmed, Nazma Begum, Rasheda Khanam, Meagan Harrison, Abdullah H. Baqui

**Affiliations:** 1 Johns Hopkins Bloomberg School of Public Health, Room E-8153, 615 N. Wolfe Street, Baltimore, MD 21205 USA; 2 K’ima:w Medical Center, 535 Airport Rd., Hoopa, CA 95546 USA; 3 Peter C. Alderman Program for Global Mental Health, HealthRight International, 14 E 4th Street, 3rd Floor, New York, NY 10012 USA

**Keywords:** Intimate partner violence, Maternal health, Mental health, Bangladesh

## Abstract

**Background:**

The purpose of this study is to assess the prevalence and associated factors of physical and sexual intimate partner violence (IPV) among married women of reproductive age in a rural population in northeast Bangladesh. In addition, we examined women’s sharing and disclosure of violence experience with others.

**Methods:**

This cross-sectional study uses data from a household survey of 3966 women conducted in 2014 in the Sylhet District of Bangladesh. Interviews were completed in respondent’s homes by trained local female interviewers.

**Results:**

Twenty-nine percent (28.8%, 95% CI 27.4–30.3%) of the women reported ever experiencing physical or sexual IPV by their spouse; 13.2% (95% CI 12.1–14.3%) reported physical or sexual IPV in the past year. Of the 13.2%, 10.1% (95% CI 9.2–11.1%) reported experiencing physical IPV and 4.6% (95% CI 4.0–5.3%) reported sexual IPV. In a combined model, the adjusted odds of having experienced physical or sexual IPV in the past year were higher for women who were raised in households with history of IPV (AOR = 4.35, 95% CI 3.26–5.80); women with no formal education (AOR = 1.76, 95% CI 1.30–2.37); women whose husbands had no formal education (AOR = 1.63, 95% CI 1.22–2.17); Muslim (AOR = 1.63, 95% CI 1.03–2.57); women younger than age 30 (AOR = 1.53, 95% CI 1.11–2.12); and women who were members of an NGO or microcredit financial organization (AOR = 1.38, 95% CI 1.04–1.82). Wealth, parity, number of household members, and pregnancy status (pregnant, postpartum, neither pregnant nor postpartum) were not associated with physical or sexual IPV after adjusting for other factors. Data on disclosure was available for women who reported experiencing physical violence in the last year; only 31.8% of victims told someone about the violence they had experienced and 1% reported to police, clerics, health workers, or a counselor altogether.

**Conclusions:**

In rural northeast Bangladesh, a high proportion of women of reproductive age experience physical or sexual IPV. Women do not often speak of these experiences, especially to anyone outside of family. Interventions aimed at preventing future IPV and addressing current IPV should focus on women who witnessed IPV in childhood, as well as younger women and less educated couples.

**Trial registration:**

This study was registered as a Clinical Trial (Identifier: NCT01702402). https://clinicaltrials.gov/ct2/show/NCT01702402

## Background

Worldwide, it is estimated that one in three women will experience physical or sexual violence at some point in their lives, most of which will occur within the context of an intimate partner relationship [1, 2]. Intimate partner violence (IPV) is a violation of human rights and carries important risks for negative impacts on physical and mental health [3]. Survivors of violence are at a higher risk for mortality, physical injury, chronic pain syndromes, depression, suicide attempts, psychosomatic disorders, gastrointestinal disorders, and reproductive health issues [4–6]. The consequences of abuse can be long lasting and the harsher and more frequent the abuse, the greater the impact on a woman’s health [7–9]. Moreover, the children of women who experience IPV are also at higher risk for poor health outcomes [10–12]. Perinatal and neonatal mortality rates are significantly higher among women who experienced IPV [13].

IPV occurs across the globe, but prevalence varies between and within countries, ranging from 15 to 71% [14]. While IPV is a global problem, it disproportionately affects low and middle-income countries (LMICs). According to recent estimates by the World Health Organization (WHO), the global lifetime prevalence of physical and/or sexual violence among ever-partnered women in LMICs is 30% [2]. For high-income countries, the prevalence is 23% [2]. Countries of South-East Asia (WHO Region) were estimated to have the highest combined lifetime prevalence (37.7%) out of all WHO Regions specified and Bangladesh is no exception [2]. Research findings from surveys conducted in Bangladesh are divergent and vary by region, with estimates of lifetime prevalence of physical IPV ranging between 42 and 76%, and estimates of annual prevalence of physical IPV to be between 16 and 67% [14–22].

Previous studies in Bangladesh have highlighted a number of factors associated with IPV against women. At a societal level, Bangladesh’s traditional patriarchy with prescriptive gender roles assign women the primary responsibility of raising children and create a woman’s economic dependence on her spouse [23]. Within classic Bangladesh patriarchy, women are married into households headed by their husbands’ fathers. Women can legally select a spouse, but this is not common. Customarily, their father or elder brother selects their future spouse. Guardianship then passes from women’s fathers to their spouses [24]. A wife’s neglect of her inherent duties or challenge to authority is interpreted as disobedience and violence by the husband is commonly accepted as justifiable punishment [24–26]. Socioeconomic advantages including wealth and educational attainment have been associated with lower prevalence of IPV [16, 21, 24, 27–29]. Women who marry at a younger age or who are presently in a marriage with dowry demands have reported increased odds of IPV [28, 30, 31]. Studies assessing the association between IPV and employment and microfinance programs for women have demonstrated mixed findings [27, 32–35]. Additional factors associated with IPV include parity, unintended pregnancy, having witnessed abuse as a child, and younger or older age [30, 36–39].

Violence against women remains largely unaddressed in many countries, including Bangladesh. Despite the large burden of IPV in Bangladesh, there is a relatively little research addressing the topic [12, 16, 17, 21, 22, 24, 26, 27, 32, 40]. Even less is known about IPV in the Sylhet Division of Bangladesh, which has the lowest levels of educational attainment, highest unemployment, ranks lowest in all measures of women’s empowerment, has consistently exhibited the highest total fertility rate, and is characterized by many other socioeconomic and health disparities [41].

IPV is often difficult to identify because it mostly occurs in private, victims are often fearful of disclosing IPV, and IPV frequently occurs in the contexts where legal systems and cultural norms do not treat these actions as a crime. Empowering established laws, such as the Suppression of Violence against Women and Children Act of 2000, to bring about justice for women in Bangladesh proves difficult [42]. Awareness of existing laws against IPV and support for punishments within the laws remains low [43]. Furthermore, criminal suits against offenders can be extremely expensive, and the women violated seldom have the resources to bring the violators to court [44, 45]. In Bangladesh, many women who experience IPV remain silent about their experience [20, 40]. Women may choose to remain silent about IPV for a number of reasons, including fear of consequences for both themselves and their children and compromising family honor [40]. Disclosure of IPV is vital for connecting victims with available resources. Moreover, understanding of disclosure patterns is needed for planning and providing context-specific programming [46].

In light of previous research and the socioeconomic disparities endemic to Sylhet, we hypothesized we would find a higher prevalence of physical and sexual IPV and that both educational attainment and wealth would be associated with less violence [16, 21, 27, 47]. In alignment with other research, we hypothesized women of a younger age, a history of their mother having been abused, and not belonging to an NGO would be associated with greater odds of having experienced IPV [24, 27, 30, 32]. The relationship between IPV and women’s participation in NGOs and micro-financing organizations is a specific area of interest. It has been argued that organizations providing microcredit loans offer economic opportunities that might protect women against violence. Others claim that financial security empowers women, which could possibly increase friction with one’s spouse and increase the risk to violence, especially in conservative areas [32]. This study aims to examine the prevalence of physical and sexual IPV, factors associated with IPV, and the disclosure patterns of married women of reproductive age in rural Sylhet, Bangladesh.

## Methods

### Study design and setting

The study data were obtained from face-to-face household interviews of 3966 women from a cross-sectional survey in the Sylhet District of Bangladesh. This was the endline survey of a quasi-experiment, the Healthy Fertility Study (HFS), conducted at the completion of the eight follow-up visits. Eight clusters, each with about 25,000 population (known as unions, the smallest administrative unit in Bangladesh, with a health center), were allocated to either intervention or control to test an integrated package of maternal and newborn health and family planning [48, 49]. The HFS was not designed to address IPV.

Beginning in December of 2007, all pregnant women identified by Community Health Workers (CHWs) in 8 unions were offered enrolment into the HFS. CHWs are locally recruited young women with an education of at least grade 10 and received 3 weeks of basic maternal and new-born health training. For all married pregnant women consenting, a baseline survey was given upon enrolment to obtain background information. Of the 4430 women recruited during pregnancy for the HFS at baseline beginning in December of 2007, 90% (*n* = 3966) were available for the endline survey in January of 2014. Due to the nature of the parent study, all women included in the endline survey had one or more live births preceding the survey.

### Assessment of variables

Trained female interviewers collected data from each study participant in her home. Socio-demographic information, such as date of birth, educational attainment, wealth (assets), and religion were collected upon enrollment into the HFS study. Wealth status was assessed at baseline by way of principal component analysis of household items. Physical and sexual IPV, the primary variables of interest in this study, were assessed during the endline survey with an instrument adapted for the 2007 Bangladesh Demographic Health Survey [22]. This modified version of the Conflict Tactics Scale was used to determine if women had experienced specific acts of physical and sexual violence. Participants were asked for a yes or no response if their husband ever did the following: (1) push you, shake you, or throw something at you; 2) slap you; 3) twist your arm or pull your hair; 4) punch you with his fist or something that could hurt you; 5) kick you, drag you or beat you up; 6) try to choke you or burn you on purpose; 7) threaten or attack you with a knife, gun, or any other weapon; 8) physically force you to have sexual intercourse with him even when you did not want to? If a participant answered “yes” to any of the aforementioned questions, they were asked if the specific violent act(s) had occurred in the last 12 months. Psychological harm (emotional abuse) was not assessed.

### Data quality assurance and ethics

Data collected in paper-based forms were entered into the database using customized data entry programs that included range and consistency checks. Detection of omissions and inconsistencies between variables were built into these programs to allow for immediate detection of simple errors. Any problems identified were reported back to the field team to ensure high quality data.

All women were enrolled in the study after informed consent. Enrolled participants were free to drop out of the study at any time and were not obligated to answer survey questions. Optimally, referral systems should be in place for women who have experienced IPV. No referral system was available in Bangladesh, and the intent of including these questions in surveys was to assess the burden and risk factors of IPV so as to design future intervention. The primary study as described was approved by the Institutional Review Board of the Johns Hopkins Bloomberg School of Public Health and the National Research Ethics Committee of Bangladesh Medical Research Council (BMRC) and was registered under clinical trial registry (Identifier: NCT01702402).

### Statistical methods

We estimated the prevalence of physical (items 1–7) and sexual IPV (item 8), as the proportion of women indicating having experienced any of the relevant violent acts. We included data for all women participating in the endline survey of 2014. Both lifetime and immediate past year estimates of each type of violence were examined.

Mixed effects logistic regression modeling was used to analyze variables associated with IPV. Mixed regression modeling takes into account community-level variables and clustering of variance that can possibly modify the relationship between the individual-level variables and IPV [32]. Factor models were constructed in a three-step sequence starting with individual factors [Model1] and hierarchically adding familial information [Model2] and membership of an NGO at the communal level [Model3]. Each model accounted for clustering to directly estimate odds ratios and standard errors [50]. Covariates including age, obstetric status, parity, education, wealth, religion, household size, historical abuse, and NGO membership were selected based on literature reviews and theoretical rationales. Analysis was conducted using the STATA (version 13) statistical package (StataCorp., 2009).

## Results

### Prevalence of physical and sexual IPV

Sample characteristics of the study participants by selected demographic variables with reported physical and/or sexual IPV are presented in Table 1. Twenty-nine percent (28.8%, 95% CI 27.4–30.3%) of women reported having ever experienced physical or sexual IPV by their husband, with thirteen percent (13.2%, 95% CI 12.1–14.3%) having experienced physical or sexual IPV in the past year. Twenty-six percent (26.2%, 95% CI 24.9–27.6%) of women reported having ever experienced physical violence and ten percent (10.1%, 95% CI 9.2–11.1%) indicated physical violence had occurred in the past year. Seven percent of women (7.2%, 95% CI 6.4–8.0%) reported sexual violence, with 5 percent (4.6%, 95% CI 4.0–5.3%) indicating sexual violence had occurred in the past year. Specific forms of violence reported by study participants are further outlined in Table 2.

**Table 1:** Frequency distributions of participants according to demographic variables with reported physical and/or sexual IPV (*n* = 3966)

Variable	Ever during marriage					During past year				
	IPV		No IPV			IPV		No IPV		
	n	%	n	%	*p* value	n	%	n	%	*p* value
**Age**					0.162					0.595
< 30 years	373	32.6	996	35.3		174	33.5	228	31.4	
30-34 years	386	33.8	957	33.9		154	31.4	951	32.7	
35+ years	384	33.6	870	30.8		169	35.1	1174	36.0	
**Pregnancy Status**					0.473					0.793
Pregnant	79	6.9	222	7.9		40	7.7	261	7.6	
Postpartum < 6 months	76	6.6	215	7.6		36	6.9	255	7.4	
Postpartum 6-12 months	89	7.8	205	7.3		44	8.4	250	7.3	
Not Pregnant nor Postpartum	899	78.7	2181	77.3		402	77.0	2678	77.8	
**Parity (total live births)**					0.053					0.545
1	239	20.9	660	23.4		122	23.4	777	22.6	
2	217	19.0	598	21.2		99	19.0	716	20.8	
3	222	19.4	528	18.7		92	17.6	658	19.1	
4+	465	40.7	1037	36.7		209	40.0	1293	37.5	
**Mother’s education**					0.000					0.000
No schooling	476	41.6	889	31.5		226	43.3	1139	33.1	
1-5 years	393	34.4	898	31.8		179	34.3	1112	32.3	
> 5 years	274	24.0	1036	36.7		117	22.4	1193	34.6	
**Husband’s education**					0.000					0.000
No schooling	554	48.5	1058	37.5		270	51.7	1342	39.0	
1-5 years	361	31.6	866	30.7		156	29.9	1071	31.1	
> 5 years	228	19.9	899	31.8		96	18.4	1031	29.9	
**Household wealth quintile**					0.011					0.005
Lowest	175	15.3	533	18.9		89	17.1	619	18.0	
Second	211	18.4	579	20.5		86	16.5	704	20.4	
Middle	264	23.1	581	20.6		121	23.2	724	21.0	
Fourth	255	22.3	551	19.5		133	25.5	673	19.5	
Highest	238	20.8	579	20.5		93	17.8	724	21.0	
**Religion**					0.003					0.052
Islam	1089	95.3	2617	92.7		498	95.4	3208	93.2	
Hindu/Others	54	4.7	206	7.3		24	4.6	236	6.9	
**Father abused Mother**					0.000					0.000
Yes	200	17.5	91	3.2		95	18.2	196	5.7	
No	695	60.8	2284	80.9		297	56.9	2682	77.9	
Do not Know	248	21.7	448	15.9		130	24.9	566	16.4	
**Members in household**					0.000					0.053
< 5	356	31.1	799	28.3		162	31.0	993	28.8	
5-9	642	56.2	1508	53.4		292	55.9	1858	53.9	
10+	145	12.7	516	18.3		68	13.0	593	17.2	
**NGO member**					0.000					0.005
Yes	187	16.4	303	10.7		84	16.1	406	11.8	
No	956	83.6	2520	89.3		438	83.9	3038	88.2	
Total	1143	28.8	2823	71.2		522	13.2	3444	86.8

**Table 2:** Reported physical and sexual IPV by study participants (*n* = 3966)

Type of violence	Ever during marriage		During past year	
	n	%	n	%
Any Physical or Sexual Abuse	1143	28.8	522	13.2
Any Physical Abuse	1040	26.2	402	10.1
Slapped	972	24.5	362	9.1
Pushed/Shaken/Thrown At	554	14.0	210	5.3
Twist Arm/Pulled Hair	266	6.7	111	2.8
Punched	365	9.2	154	3.9
Kicked/Dragged/Beaten Up	356	9.0	154	3.9
Choked/Burned	59	1.5	25	0.6
Threatened with Knife/Gun	30	0.8	14	0.4
Forced Sex	286	7.2	183	4.6
One Type of Violence	403	10.2	201	5.1
2-3 Types of Violence	452	11.4	186	4.7
4-5 Types of Violence	229	5.8	77	1.9
6+ Types of Violence	59	1.5	58	1.5

Note: Some women experienced more than one type of violence. Of the 522 women who experienced either physical or sexual IPV in the past year, 402 experienced physical IPV, 120 experienced sexual IPV, and 63 experienced both types of IPV

### Factors associated with physical and sexual IPV

Women who witnessed their father hit or beat his wife (the mother of the interviewee) were significantly more likely to have experienced IPV in the past year (AOR = 4.35, 95% CI 3.26–5.80) (Table 3). The odds of having experienced physical or sexual IPV in the past year were significantly higher for women with no formal education (AOR = 1.76, 95% CI 1.30–2.37) or 1–5 years of education (AOR = 1.54, 95% CI 1.17–2.02) as compared to women with 6 or more years of education. Furthermore, women whose husbands had no education were more likely to report having experienced physical or sexual IPV in the past year than women whose husbands had 6 or more years of education (AOR = 1.63, 95% CI 1.22–2.17). Study participants who self-identified as Muslim were significantly more likely to report having experienced physical or sexual IPV in the past year than non-Muslim women (AOR = 1.63, 95% CI 1.03–2.57). Women younger than 30 years of age experienced a relatively higher prevalence of physical or sexual IPV in the past year (AOR = 1.53, 95% CI 1.11–2.12) in comparison to those 35 years of age and older. Members of an NGO or financial organization were more likely to have experienced IPV in the past year (AOR = 1.38, 95% CI 1.04–1.82).

**Table 3:** Odds of physical or sexual violence by husband in the past year by selected variables (crude and mixed effects model)

Variable	Crude OR				Adjusted model OR			
	OR	*p* value	95% CI		AOR	*p* value	95% CI	
**Age**								
< 30 years	1.05	0.67	0.84	1.31	1.53	**0.01**	1.11	2.12
30-34 years	0.98	0.79	0.77	1.22	1.20	0.16	0.93	1.55
35+ years	Reference				Reference			
**Pregnancy Status**								
Pregnant	1.04	0.84	0.73	1.47	1.00	0.99	0.69	1.44
Postpartum < 6 months	0.97	0.86	0.67	1.39	0.97	0.88	0.66	1.42
Postpartum 6-12 months	1.18	0.35	0.84	1.65	1.19	0.34	0.83	1.71
Not Pregnant nor Postpartum	Reference				Reference			
**Parity (total live births)**								
1	Reference				Reference			
2	0.89	0.42	0.67	1.18	0.82	0.20	0.60	1.11
3	0.89	0.45	0.67	1.19	0.82	0.25	0.59	1.15
4+	1.04	0.73	0.82	1.32	0.88	0.46	0.62	1.24
**Mother’s education**								
No schooling	2.09	**0.000**	1.64	2.66	1.76	**0.000**	1.30	2.37
1-5 years	1.07	**0.000**	1.33	2.18	1.54	**0.002**	1.17	2.02
> 5 years	Reference				Reference			
**Husband’s education**								
No schooling	2.19	**0.000**	1.71	2.80	1.63	**0.00**	1.22	2.17
1-5 years	1.60	**0.001**	1.22	2.09	1.23	0.16	0.92	1.64
> 5 years	Reference				Reference			
**Household wealth quintile**								
Lowest	Reference				Reference			
Second	0.83	0.24	0.60	1.14	0.71	**0.04**	0.51	0.98
Middle	1.12	0.44	0.84	1.51	0.94	0.70	0.69	1.29
Fourth	1.36	**0.04**	1.01	1.82	1.06	0.71	0.77	1.46
Highest	0.87	0.37	0.63	1.19	0.73	0.06	0.52	1.02
**Religion**								
Islam	1.53	**0.05**	0.99	2.38	1.63	**0.04**	1.03	2.57
Hindu/Others	Reference				Reference			
**Father abused Mother**								
Yes	4.56	**0.000**	3.44	6.02	4.35	**0.000**	3.26	5.80
No	Reference				Reference			
Do not Know	2.26	**0.000**	1.79	2.85	2.20	**0.000**	1.74	2.79
**Members in household**								
< 5	Reference				Reference			
5-9	0.98	0.87	0.80	1.21	0.99	0.93	0.80	1.23
10+	0.72	**0.03**	0.53	0.97	1.19	0.32	0.62	1.16
**NGO member**								
Yes	1.44	**0.01**	1.10	1.88	1.38	**0.03**	1.04	1.82
No	Reference				Reference			

In comparison to women of the lowest wealth quintile (poorest), women in the second lowest quintile (second poorest) were less likely to report having experienced physical or sexual IPV by their spouse (AOR = 0.71, 95% CI 0.51–0.98). However, no higher wealth quintiles were significantly different from the poorest. Hence, no clear trend between wealth quintiles and IPV was detected. No significant association was detected between parity and IPV. Moreover, women who were pregnant or postpartum (up to 1 year) did not report significant differences in having experienced physical or sexual IPV in the past year. Past year prevalence of physical or sexual IPV was not significantly different between women of differing household sizes (number of persons).

### IPV antecedents, reporting, and perceptions

Among women who reported physical IPV in the past year (*n* = 402), the majority (65.2%) reported that nothing in particular prompted the physical abuse by spouses (Table 4). The most common responses given by women to the question of why physical violence in the past year occurred were financial crisis (22.9%), disobeying their spouse or elder (21.4%), neglecting chores (19.7%), and refusing sex (14.8%).

**Table 4:** Women’s perceived reasons for physical abuse by husband (*n* = 402)

Perceived reasons for abuse	n	%
Without Reason	262	65.2
Financial Crisis	92	22.9
Disobeyed Husband/Elder	86	21.4
Neglected House Chores	79	19.7
Refused Sex	57	14.2
Bacause Husband Unemployed	29	7.2
Envy or Malice	27	6.7
Wife Suspects Infidelity	21	5.2
Food Crisis	18	4.5
For Dowry	15	3.7
Demand of Money From My Family	14	3.5
Went Out Without Permission	5	1.2
Husband Suspects Infidelity	5	1.2
Husband’s Drug/Alcohol Use	5	1.2
Other	4	1.0

Approximately one third (31.2%) of women who reported physical IPV in the past year (*n* = 402) told someone (Table 5). When victims did tell someone about physical abuse, they most often told their parents, in-laws, neighbors, siblings, and/or extended family. Local leaders, police, clerics, and health workers were seldom told. Nearly 90% of women who had been physically or sexually abused in the past year felt their husband treats them fairly.

**Table 5:** Who physically abused (in past year) women told about experiences of IPV (*n* = 402)

Person told	n	%
Anyone	128	31.2
Mother/Father	61	15.2
Mother-In-Law	41	10.2
Neighbor	37	9.2
Brother/Sister	26	6.5
Father-In-Law	20	5.0
Other Relative	19	4.7
Aunt/Uncle	15	3.4
Children	7	1.7
Local Leader	7	1.7
Friends	3	0.7
Police	2	0.5
Cleric	2	0.5
Doctor/Healthworker	1	0.2
Counselor	1	0.2
Other	1	0.2

## Discussion

Our study documented a lifetime (28.8%) and past year (13.2%) prevalence of reported physical or sexual violence among married women of reproductive age in rural Sylhet District of Bangladesh. The rates are similar to globally observed rates, but lower than studies conducted in Bangladesh [14–21, 51]. According to the Bangladesh Demographic Health Survey, 2007 (BDHS, 2007), among all 8 administrative divisions of Bangladesh, Sylhet Division had the lowest reported prevalence (41.7%) of lifetime physical or sexual IPV [22]. The finding was surprising considering that Sylhet faces the most socioeconomic and health disparities among all divisions [41]. We have observed an even lower rate than documented in BDHS. Sampling and data collection methodology was different in our study than the BDHS, which may explain why our prevalence findings for Sylhet were approximately 13% lower than that reported by the BDHS. For the BDHS, either husbands or wives were interviewed in order to obtain estimates of IPV in the Sylheti population. Moreover, the BDHS had a comparatively smaller sample size of only 283 participants. Also, all women in our study population had young children, which could possibly be a protective factor. Furthermore, data collection in our study took place as part of an intervention. Participants may have given a more socially desirable answer for fear of not receiving services or upsetting people that provide services. Abused women in Bangladesh are often silent about experiences of violence for reasons including fear of repercussions from the abuser and compromising family honor, which can also influence study findings [40].

Women whose mother had been abused by her husband (father of interviewee) reported an astounding four-fold the odds of having experienced physical or sexual violence in the past year. It may be that fathers select a spouse for their daughter who shares common behaviors and attitudes, such as strict gender roles and justification for abuse when crossed [24]. In addition, cultural, economic, and educational similarity between families of arranged marriages might result in a transfer of patterns of abuse to the next generation. An alternative explanation of this strong relationship could be that women who are willing to admit to having been abused are also willing to admit their mother had been abused, thus strengthening the detected association.

Our findings that lower education level of both the wife and her husband were associated with experiencing higher odds of physical or sexual IPV in the past year is consistent with earlier findings from Bangladesh [16, 31]. Men with perceived power, such as a higher degree of education, may have less need to assert their dominance via IPV [52–54]. In addition, communication or cognitive skills obtained from greater educational attainment may contribute to better conflict resolution within marriage [47]. Moreover, school programming and relationships may influence attitudes towards the acceptability of violence in both males and females and thereby reduce IPV [55].

Muslim women reported a higher prevalence of physical or sexual violence within marriage in comparison to women of other religions (predominantly Hindu). Our findings in rural Bangladesh were similar to Naved’s, who postulated that the difference in IPV might be explained by Muslim women traditionally having less mobility and independence than Hindu women in Bangladesh, which limits access to people and resources [27]. Specific reasons for the association between IPV and religion are inconclusive without further qualitative research.

In accord with other studies from Bangladesh, we found married women of younger ages experienced higher odds of IPV in the past year in comparison to older women of reproductive age [27, 30]. In Bangladesh, younger women are more likely to have an arranged marriage and have lower social status [56]. Though our data was not longitudinal, based on responses by women of different pregnancy status, findings did not suggest a reprieve of physical or sexual violence during pregnancy or postpartum as has been indicated by other research in Bangladesh [19, 31]. Moreover, we did not find a significant relationship between parity or number of household members and IPV.

It may seem counterintuitive that women who were members of an NGO or microfinancing organization had comparatively higher odds of having experienced IPV in the past year, but perhaps the association could be explained by the explicit goal of NGOs to target the most vulnerable populations and lead to selection bias [57]. It is difficult to interpret this finding in a cross-sectional study, where risks for reverse causality exist. For example, it is possible that women who experience IPV more commonly seek out NGOs for support. However, since our analysis did adjust for levels of wealth, education, and union (smallest administrative unit) membership, this finding is worthy of further consideration. One element that has been suggested for this particular context is that NGOs and microfinancing institutions in Bangladesh often include “women’s empowerment” initiatives and microfinance programs for women. Such initiatives could possibly upset the balance of power in relationships and unintentionally lead to a period of increased violence and tension [25, 32, 58–60]. Based on recent quantitative and qualitative research, Murshid suggests that microfinance programming in Bangladesh should be coupled with IPV screening and intervention [33–35].

Poverty has been noted as a common risk factor for IPV [21, 27]. Our study did not yield a significant association when examined by wealth quintile based on household assets. Interestingly, while wealth was not associated with IPV, financial crisis was given as a reason for having experienced physical or sexual violence by 23% of women who self-identified as having experienced violence in the past year. Perhaps, given that the majority of women in Bangladesh experience poverty, financial tension is a common occurrence across all wealth strata thereby reducing differentiation across wealth quintiles. Particular financial ups and downs within a wealth stratus may then be a more accurate predictor of IPV than the level of wealth per se. Alternatively, our methodology of accounting for wealth through household assets, which is a common method in developing countries, might not discriminate people into appropriate socioeconomic categories where poverty is widespread or be sensitive to seasonal trends or fluctuations in income that could give rise to stress or conflict. Additionally, household assets do not provide insight as to which household members are contributing funds, which might influence power dynamics and IPV. It could be true that wealth is not associated with IPV in our sample of women [16].

Disobedience, neglect of household chores, and refusal of sex were commonly cited as antecedents of physical abuse, which can be interpreted as typical characteristics of stringent gender roles within a patriarchal system [24, 26, 61, 62]. We found that 65% of women who had experienced violence in the past year indicated that they had experienced abuse without a particular antecedent. In a sample of 496 ever-married Bangladeshi women, Yount and colleagues (2013) found that women (38–68%) justified abuse by the husband when the woman’s transgression was depicted as willful, especially disobeying elders in the marital family (68%), neglecting the children (65%), and arguing with the husband (59%). Conversely, relatively fewer women (1–8%) justified wife hitting or beating when her transgression, as perceived by the perpetrator, was depicted as unintended [24].

Approximately one in three women (31.2%), who experienced IPV, shared their experience with others. Considering the possibility that some abused women interviewed in our study may have withheld their experiences with IPV, it appears that most women in our study population experiencing IPV are never assisted in any way. Sharing with or pursuit of formal avenues (police, counselors, health workers, etc.) for assistance with IPV was virtually non-existent (< 1%).

Our study is not without limitations. Due to the cross-sectional nature of this study, factors associated with IPV may not be causal. Our statistical modeling for factors did not include all possible covariates. For example, it is common for Sylheti men to migrate for work, which subsequently reduces the probability of IPV. While the unions surveyed in this study were typical of rural Bangladesh, characteristics did vary by union and generalizability of the results may be limited. The possibility of reporting bias in our study is of concern, particularly given that we found a lower prevalence of IPV than other studies conducted in Bangladesh. IPV could have been underreported due to many factors, such as fear of repercussions, shame, or loss to follow-up of women most at risk for abuse. Our study survey was conducted years after a live birth, thereby excluding newlyweds and women without children.

Though IPV affects a large proportion of the women directly and everyone indirectly, minimal research and few resources have been dedicated to addressing the enormous problem in Sylhet, Bangladesh. Our study documents that a large proportion of women of reproductive age in rural Bangladesh suffers from the atrocities of IPV. In 1993, the United Nations General Assembly declared, “We must ensure zero tolerance of violence against or exploitation of women and girls [63].” The Millennium Development Goals (MDGs) and newly established Sustainable Development Goals (SDGs) stress gender equality, women’s empowerment, and improvement of maternal health.

The need for prevention of IPV and efforts to reduce IPV where it occurs, are clear. Generational transfer of IPV is evident and demonstrates that families, in and of themselves, are not likely to break the cycle of abuse automatically. Based on a 2014 review of interventions to reduce violence against women in LMICs, interventions designed to prevent IPV should be multifaceted, address risk-factors, and engage many stakeholders [64]. Specifically, for Sylhet, interventions should target women with a history of parental IPV, as well as younger, and lesser-educated couples. Ideally, primary prevention should exist prior to marriage. Those who had obtained little education were most probable for perpetrating violence (husbands) and experiencing violence (wives). Limiting primary prevention to school settings alone would neglect vast majority of at-risk populations. Multisectoral programs enabling community engagement and participatory approaches to address cultural attitudes and behaviors towards IPV have been found to be effective [64]. In our study, most women who experienced IPV did not tell anyone and pursuit of formal avenues for help was highly unlikely. Addressing the multiple stakeholders and equipping both informal and formal avenues of assistance can lead to positive change [64]. Social and/or economic interventions to reduce IPV in LMICs have been associated with decreases in controlling behaviors by partners, relationship quality improvements, and economic gains [65]. Based on our study finding that NGO membership was a risk factor for IPV, caution is warranted when conducting interventions. Future research on effective IPV prevention and reduction strategies is urgently needed.

## Data Availability

De-identified data is available. The datasets used and/or analyzed during the current study are available from the corresponding author upon reasonable request.

## Abbreviations

IPV: Intimate partner violence
WHO: World Health Organization
NGO: Non-Governmental Organization
HFS: Healthy Fertility Study
CHW: Community health worker
BDHS: Bangladesh Demographic Health Survey
